# Evaluating suicide prevention gatekeeper training designed to identify and support people from asylum-seeking and refugee backgrounds

**DOI:** 10.1186/s12889-024-20304-3

**Published:** 2024-10-25

**Authors:** Steven MacDonald Hart, Erminia Colucci, Lisa Marzano

**Affiliations:** 1 The Suicide Care, Prevention, and Research Initiative, The Department of Medical & Clinical Psychology, Uniformed Services University of the Health Sciences, Bethesda, MD United States of America; 2 The Department of Psychology, Middlesex University, Hendon, London, England

**Keywords:** Suicide Prevention, Gatekeeper training, Asylum-seekers, Refugees, Tailored, Forced Migration, Marginalized populations

## Abstract

**Background:**

Suicide-related behaviours and individual risk factors for suicide differ between ethnicities and demonstrate additional variation based on voluntary and forced migration. People forcibly displaced by violence and conflict, such as those seeking asylum and refugees, are likely to face stressors that can increase suicide risk. Research into evidenced-based suicide prevention strategies among people from asylum-seeking and refugee backgrounds is scarce. However, early, contextually-appropriate, identification and intervention may be a promising way to facilitate support for people in these groups. This research proposes that a contextually-responsive gatekeeper training is an appropriate strategy to increase the identification and support for people from asylum-seeking and refugee backgrounds.

**Methods:**

The present article relates to the statistical findings of a larger mixed-method study used to validate and refine a contextually-responsive gatekeeper training program. The qualitative results of this research will be published separately. The outcome measures – knowledge about suicide in multicultural contexts, attitudes towards suicide and prevention, and self-efficacy to intervene were measured quantitatively, adopting a similar pre- and post-training procedure used in previous training evaluations. Using Generalised Estimating Equations, statistical comparisons were made between three identical self-report surveys completed by participants across three consecutive time points – pre-training, immediately post-training, and three months following training completion – known in this investigation as time-point zero (T0), time-point one (T1), and time-point two (T2). Lastly, during the T2 follow-up, additional open-ended questions were included to understand which areas of training they feel prepared them effectively and how the program could have better prepared them to intervene.

**Results:**

A total of 28 participants took part in the study. Quantitative analysis indicated the program’s capacity to exert a significant favourable and lasting influence on knowledge about suicide and self-efficacy to intervene. In addition, follow-up measurements suggest that the content delivered to participants transferred effectively into real-world suicide prevention behaviours.

**Conclusions:**

Findings suggest that tailored suicide prevention training can have a significant influence on knowledge about suicide in multicultural contexts, self-efficacy to intervene in a crisis, and that course content translates effectively into real-world suicide prevention behaviour. Modifying training practices, based on feedback from contextually-experienced attendees, appears to be a pivotal factor in promoting the support of people from asylum-seeking and refugee backgrounds.

## Background

Suicide is a major public health concern, claiming the lives of at least 720,000 people each year [1]. The epidemiology of suicide is characterised by vast regional variation, with low- and middle-income countries (LMICs) representing 73% of the World’s annual suicide rate [1]. Suicide-related behaviours and individual risk factors for suicide vary between cultures and ethnicities [2–4]. International migration, whether forced or voluntary, has been associated with further fluctuations in suicide-related behaviour [5]. Depending on the country of origin and the individual circumstances of the person migrating, people may have pre-existent higher or lower suicide risk that accompanies them when relocating [6]. An argument that is generally agreed upon, however, is that people from asylum-seeking and refugee (AS&R) backgrounds are susceptible to a plethora of vulnerabilities [7, 8], which increase their likelihood of experiencing hopelessness, suicidal thoughts, and exhibiting suicide-related behaviours [9].

Worldwide, at the end of 2023, there were a reported 110 million individuals displaced due to violence, conflict, and persecution [10]. People fleeing war and persecution are particularly vulnerable to stressors that can increase their risk of suicide through a high probability of traumatic pre-migration experiences, life-threatening journeys to seek refuge, and often living in destitution upon arrival in a host country [11, 12]. People from AS&R backgrounds are likely to have been exposed, directly and vicariously, to potentially traumatising circumstances, including extreme physical violence and socio-political persecution [13]. For some, the migration process can accompany highly distressing experiences, such as life-threatening means of transport, sexual exploitation, physical violence, malnourishment, and detention [7, 12, 14]. Even upon arrival in a host nation, people from AS&R backgrounds, and migrants in general, can be subject to racism, discrimination, acculturation stress, and social and economic marginalisation [15–17]. In addition, for those seeking asylum in particular, the distress associated with the uncertainty and delays during visa application processing, coupled with restricted rights to employment, financial support, and access to public services, can instill perceptions of ‘lethal hopelessness’ and increased suicidal intent [18, 19].

Accessing public and specialised healthcare can be problematic for people from AS&R backgrounds, relative to native-born residents, as they face a heightened likelihood of encountering structural barriers to care [20]. Such structural barriers can include a lack of knowledge on accessing and navigating health care, language difficulties, and inadequate identity and immigration documentation [15, 21, 22]. Barriers to accessing support also include personal reluctance due to distrust of people in positions of authority and fear of breaches in confidentiality [23, 24] or culturally embedded stigmatising beliefs that can result in negative attitudes to seeking mental health support [25]. Despite their susceptibility to stressors that can lead to suicide-related behaviour, coupled with their low likelihood of engaging in formal help-seeking behaviours, research focused on suicide prevention strategies to support people from these groups is scarce [26]. Promisingly, however, international research into culturally-informed, suicide prevention gatekeeper training (GKT) tailored to meet the needs of its target population is growing, including for indigenous populations in Australia [27], youths in Guyana [28], children and young people in conflict-affected areas in Syria [11], and people from AS&R backgrounds in Australia [9]. To date, however, there is a scarce amount of systematic research into culturally informed, evidence-based GKT programs to support people from AS&R backgrounds in Europe.

A gatekeeper (GK), in the context of suicide prevention, broadly describes a person uniquely positioned to identify suicide risk because of their regular engagement with a large number of people [29]. GKs are divided into emergent and divergent categories [30]. Designated GKs operate at specialised levels, such as counsellors, social workers, or medical professionals. Emergent GKs are community members whose roles or relationships may increase their likelihood of interacting with a person at risk of suicide [30]. GKT refers to a body of educational programs that provide GKs with the knowledge and skills to identify people at risk for suicide, engage, and appropriately refer them to further support [4, 31]. GKT programs have demonstrated particular effectiveness when implemented as part of a systematic effort to prevent suicide [32, 33] and have been incorporated into numerous community-based prevention initiatives [34].

Knowledge about suicide is an asset for gatekeepers, as it equips them to identify the possible warning signs of suicide and the ability to allocate appropriate intervention strategies [29]. Attitudes toward suicide prevention encapsulate, though are not limited to, perceptions of whether it is possible to prevent suicide, whether or not an intervention is appropriate, and whether seeking mental health assistance should be sought personally [29, 35]. Potential caregivers may inhibit themselves from intervening in a person’s crisis by perceiving that it is inappropriate or because they feel it is not their responsibility to do so [29].

Self-efficacy (SE) to intervene refers to one’s belief that they can identify the warning signs for suicide, care for a person at risk, and effectively refer them to seek further support for their suicidal thoughts [29]. Exerting a positive influence over these constructs is argued to promote the likelihood of intervention and referral behaviours [31]. Existing GKT has demonstrated the ability to positively influence attendees’ knowledge about suicide [36], attitudes towards prevention [11, 37], and self-efficacy to intervene [38].

The current study proposes that GKT, given the strategy’s ability to be tailored to meet the unique requirements of its target population [4, 30], is an appropriate strategy to increase the identification and support of people from asylum-seeking and refugee backgrounds in the UK. The GKT program around which this research is centred is named Suicide Intervention First Aid (SIFA), refered to henceforth as Suicide Intervention Training). Suicide Intervention Training was built on the foundation of knowledge provided by the Suicide First Aid Guidelines^1^ for Supporting People from an Immigrant or Refugee Background [39], is an online program comprising nine hours of material, equally distributed into three-hour segments, and delivered across three consecutive weeks. This research sought to validate and refine the program as a contextually responsive suicide prevention gatekeeper training program (GKT) that can enable gatekeepers’ ability to identify and support people from asylum-seeking and refugee backgrounds experiencing a suicidal crisis.

## Statistical hypotheses

### *H*1_A_

Immediately following training, there will be a significant increase in mean scores for knowledge about suicide in multicultural contexts, attitudes towards suicide and prevention, and self-efficacy to intervene in a crisis.

### *H*2_A_

The short-term, post-training mean scores for knowledge about suicide in multicultural contexts, attitudes towards suicide and prevention, and self-efficacy to intervene in a crisis will reduce over three months while remaining significantly higher than baseline.

## Methods

### Ethical approval

The Psychology Research Ethics Committee approved the current research at Middlesex University (Project ID: 7614).

### Program Development

Suicide Intervention Training was created on the foundation of knowledge provided by the Suicide Guidelines for supporting people from immigrant or refugee backgrounds [39]. The course is a remote learning program that takes nine hours to complete, and consists of three core modules: understanding suicide; which includes epidemiology of suicide and definition of terms; the warning signs for suicide; and referral pathways and processes. The program is split into three segments, each lasting three hours, and delivered across three consecutive weeks. Each of the three core modules comprised didactic lectures that were supplemented with interactive discussions and roleplay activities (e.g., practising asking about thoughts of suicide, engaging in therapeutic risk assessments, and creating collaborative safety plans).

### Participants and recruitment

 Originally, Suicide Intervention training was intended to be delivered in person. However, the program was modified to be delivered remotely due to social-distancing measures implemented to reduce the spread of COVID-19. Participants who attended training were a self-selected volunteer sample affiliated with a non-profit charity that provided practical aid to people from asylum-seeking and refugee backgrounds across the UK, France, and Belgium. The charity offers support by providing clothes, bedding, food, medical care, and legal, social, and educational assistance. Participants were recruited through an invitational email sent to all employees and volunteers via the charity’s online database.

Following participants’ initial interest in attending training (*N* = 37), complete program details were provided through a participant information sheet, including the course aims, delivery dates and times, research structure and requirements should they choose to participate. The average age of the sample was 37.86 years (*SD* = 13.032), with the youngest GK being 19 and the most senior 64. Most of those who attended the training were female, representing 97% of the sample (*n* = 27). 21% of GKs were employees (*n* = 6), and 78% were volunteers (*n* = 22). Between the sample, their total duration of experience supporting people seeking asylum and from refugee backgrounds equalled 25.1 years.

Training was delivered four times over Zoom between March 2021 and February 2022. From the initial delivery of training onwards, a list of potential participants was compiled (through their request) for those who could not attend scheduled dates for a given round of training so that they could be offered the opportunity to attend subsequent dates.

### Outcome measures

Participants were asked to complete a basic demographic questionnaire. The questionnaire collected information about nationality, cultural affiliation and/or ethnicity, gender, age, occupation, role within the charity, and whether they had previous formal mental health or suicide prevention training. Knowledge about suicide in multicultural contexts was measured using a scale consisting of 48 potential warning signs for suicide for people from migrant or refugee backgrounds (α = 0.83) (25 of which were included in the Suicide Guidelines for supporting people from immigrant and refugee backgrounds). Participants were asked to rate the items they believed to be accurate. Correct answers, whether ‘accepted’ or ‘rejected’, were coded with a score of one, and an incorrect answer was assigned zero so that a composite score could be computed (e.g., 21 out of 25). Importantly, gatekeepers were informed that ‘accepted’ warning signs might signal imminent concern; however, ‘rejected’ warning signs should not be dismissed.

Participants’ attitudes towards suicide and suicide prevention were measured using an adapted version of the Attitude towards Youth Suicide Scale (AtYS; [35]). The AtYS is a 21-item survey designed to understand cross-cultural variations in attitudes towards youth suicide (α = 0.6). Research demonstrates that there is no particular gold standard approach to measuring attitudes surrounding suicide [40]. Therefore, the AtYS was implemented because of its previous application in cross-cultural research [35], as it was deemed better suited to the present research than a generic scale. The AtYS was modified to align with the current study by replacing all references of ‘youth’ with persons. Gatekeepers’ responses were rated on a 5-point Liker scale (i.e., 1 = Strongly Agree, 2 = Agree, 3 = Neither Agree nor Disagree, 4 = Disagree, and 5 = Strongly Disagree), and negatively worded items were reverse coded. Lower scores, therefore, represented more positive attitudes. Participants’ self-efficacy to intervene in a suicidal crisis was measured using an adapted scale that was originally designed to measure confidence to intervene in a suicidal crisis among rail police [41]. The scale comprises three items, including “I feel I can accurately identify situations where a person is at risk of suicide,” “I know how to approach people at risk of suicide,” and “I know how to refer people at risk of suicide to the most appropriate services for their needs.” The response structure was a five-point Likert scale, identical to the AtYS, with a lower response indicating greater self-efficacy. The self-efficacy scale demonstrated a low internal consistency (α = 0.44); likely due to the tendency of Cronbach’s alpha to underestimate consistency when a scale consists of fewer than 10 items [42].

### Procedure

The current article relates to the statistical findings of a larger study, utilising a mixed-method, action research approach to validate and refine a contextually-responsive gatekeeper training program. To assess the impact of training on knowledge about suicide in multicultural contexts, attitudes towards suicide and prevention, and self-efficacy to intervene in a crisis, participants were asked to complete three online surveys. The three surveys were each created, distributed, and completed through Qualtrics (https://wwww.qualtrics.com). The initial two surveys of each training round were distributed during training through the chat feature of Zoom online video conferencing software, immediately before (T0) and after (T1) training. The third survey was emailed to participants three months after program delivery (T2).

### Data analysis

Study surveys took 20–30 min to complete, and from those who initially attended training (*N* = 32), changes in work/volunteering commitments led to attrition during the program’s delivery (*N =* 4). These participants had only completed the T0 survey, so their scores were removed from the analysis. Following removal, (*N =* 28) were available for the short-term analysis of training effects (i.e., comparing T0 with T1). Finally, at three-months follow-up, the study experienced a 50% (*N* = 14) attrition rate between (T1 and T2). Follow-up emails were sent to those who did not respond to the email containing the T2 survey link; however, the PI received no further response. Originally, the intention was to assess the study hypotheses using a within-groups repeated measures analysis of variance (Repeated Measures (RM) ANOVA). However, because the study experienced large attrition rates between T1 and T2, it was decided that an RM ANOVA would be insufficient to handle this missing data. Instead, the analysis utilised Generalised Estimating Equations (GEE) to handle missing data and preserve statistical estimates [43]. Suicide Intervention Training represented the independent variable; knowledge about suicide in multicultural contexts, attitudes towards suicide and suicide prevention, and self-efficacy to intervene in a crisis were dependent variables, and the within-subjects factor was time, with three levels (i.e., timepoints zero = T0, one = T1, and two = T2). For all outcome variables, differences in the mean scores between the three time points (T0, T1, and T2) were evaluated by fitting linear GEE models factoring in the time point of assessment. Maximum likelihood was selected for parameter estimation, and the structure of the working correlation matrix was specified as “independent” with a robust estimator. Wald tests were computed to determine the effects in all cases. Post-hoc Bonferroni-corrected comparisons were conducted to examine the effects further.

## Results

Before conducting the inferential analysis, normality was assessed, and no significant violations emerged (Shapiro-Wilk, all *p* > .05). As shown below in Table 1, the average age of the sample was 37.86 years (*SD* = 13.032), with the youngest GK being 19 and the most senior 64. Most of those who attended the training were female, representing 97% of the sample (*n* = 27). 21% of GKs were employees (*n* = 6), and 78% were volunteers (*n* = 22). Between the sample, their total duration of experience supporting people seeking asylum and from refugee backgrounds equalled 25.1 years.

**Table 1:** Sample characteristics (*N* = 28)

	*n*	%	M	SD
Age (years)	28	**-**	37.86	12.03
Female gender	27	97		
**Charity Affiliation**				
Employee	6	21		
Volunteer	22	78		
**Experience**				
Combined experience supporting people from asylum seeker and refugee backgrounds (years)	25.1	-		
Previous mental health and/or suicide prevention training	8	28.6		
**Nationality**				
British	21	75		
British/French	1	3.6		
British/Italian	1	3.6		
Cypriot	1	3.6		
French	2	7.1		
Polish	1	3.6		
Slovak	1	3.6		
**Cultural Affiliation and/or Ethnicity**				
Algerian, French, British	1	3.6		
Greek Cypriot	1	3.6		
British/Indian	1	3.6		
Pakistani	1	3.6		
Spanish, French, German	1	3.6		
White British	19	67.9		
White European	4	14.6		

### Knowledge about suicide in multicultural contexts

**Fig. 1:**
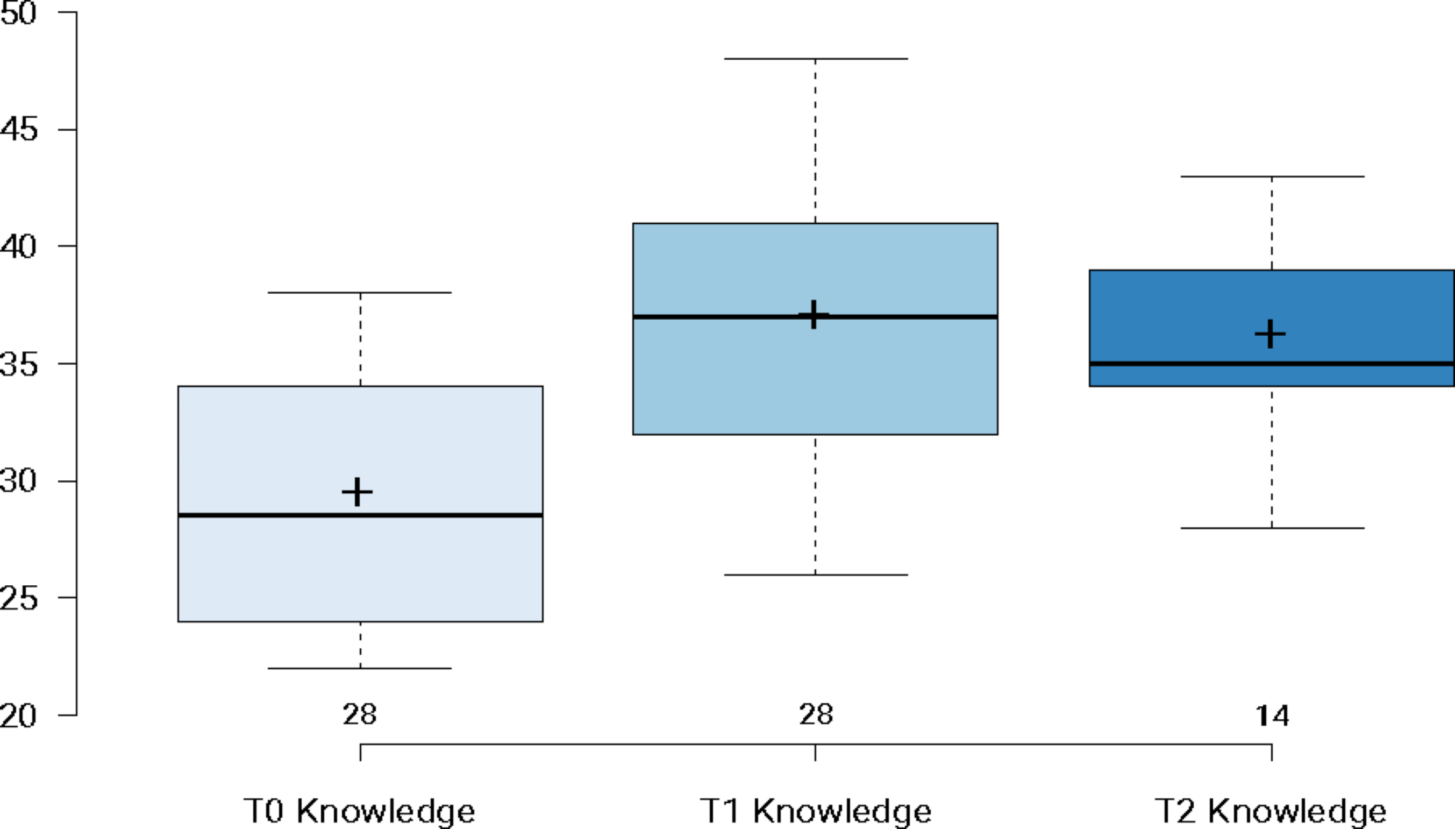
Changes in knowledge about suicide in multicultural contexts. **Note:** Boxplots illustrating participants’ Knowledge About suicide in Multicultural Contexts at T0 (*N* = 28), T1 (*N* = 28) and T2 (*N* = 14). Horizontal lines illustrate median scores; and + symbol illustrates mean scores

As demonstrated below in Fig. 1, participants’ knowledge about suicide in multicultural contexts was assessed across three time points (T0, T1, and T2). Wald tests were computed to determine the significance of the main effects (Waldχ^2^ = 39.576, *p* < .001). Bonferroni-corrected pairwise comparisons indicated that there was a significant increase in mean scores from T0 to T1 (*p* < .001), no significant change from T1 to T2 (*p* = 1.0), and a significant increase at T2 relative to T0 (*p* < .001). These results support the study’s hypothesis, indicating that training immediately improved gatekeepers’ knowledge about suicide in multicultural contexts, which not only remained above baseline three months after completion but demonstrated no statistical deterioration between the two post-training measurements.

### Self-efficacy to intervene in a crisis

**Fig. 2:**
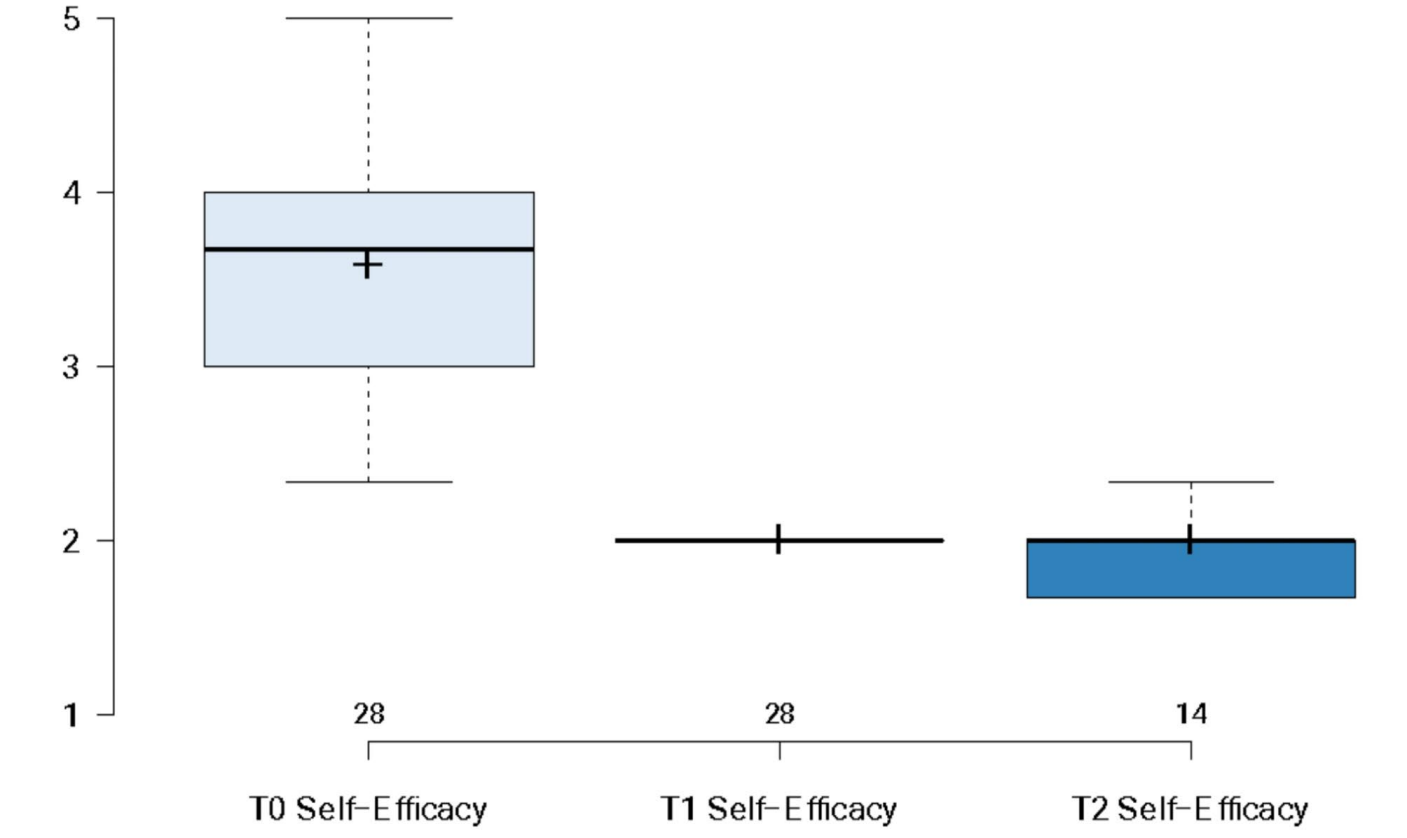
Changes in self-efficacy to intervene in a crisis. **Note**: Boxplots illustrating participants’ self-efficacy to intervene in a crisis at T0 (*N* = 28), T1 (*N* = 28) and T2 (*N* = 14). Horizontal lines illustrate median scores; and + symbol illustrates mean scores

As shown in below in Fig. 2, participants self-efficacy to intervene in a crisis was measured across three time points (T0, T1, and T2). Wald tests were computed to determine the significance of the main effects (Waldχ^2^ = 129.882, *p* < .001). Bonferroni-corrected pairwise comparisons indicated a significant increase from T0 to T1 (*p* < .001), no significant change from T1 to T2 (*p* = 1.0), and a significant increase at T2 relative to T0 (*p* < .001). These results suggest that training was able to exert a significant increase in GKs’ baseline scores in their self-efficacy to intervene in a crisis immediately following the completion of training, which was able to be maintained three months later.

### Attitudes toward suicide and suicide prevention

**Fig. 3:**
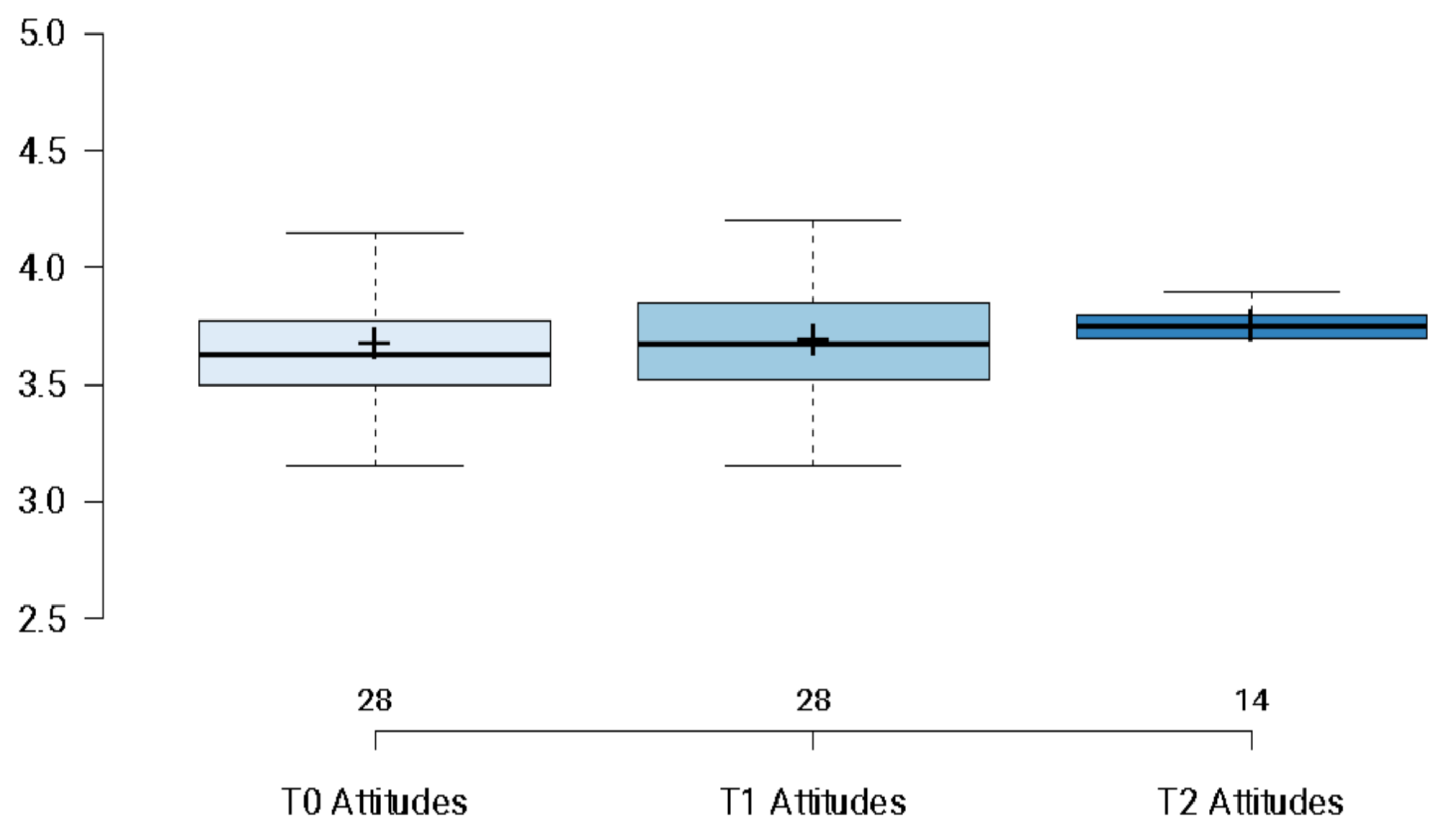
Changes in attitudes towards suicide and suicide prevention. **Note**: Boxplots illustrating participants’ attitudes towards suicide and suicide prevention at T0 (*N* = 28), T1 (*N* = 28) and T2 (*N* = 14). Horizontal lines illustrate median scores; and + symbol illustrates mean scores

As depicted in below in Fig. 3, participants attitudes towards suicide and suicide prevention were assessed across three time points (T0, T1, and T2). Wald tests were computed to determine the significance of the main effects (Waldχ^2^ = 3.480, *p* < .001). No significant increase was observed from T0 to T1 (*p* = 1.0). Further, there was no significant change from T1 to T2 (*p* = 1.0) and no significant difference at T2 relative to T0 (*p* = 1.0). Contrary to the study’s hypothesis, gatekeepers’ attitudes towards suicide and prevention remained stable across time points, suggesting that training could not affect this outcome measure.

#### Post-training suicide prevention behaviour

From the number of GKs who attended training from start to finish (*N* = 28), *n =* 14 (53.57%) completed the three-month follow-up survey (i.e., T2). During those three months, *n* = 14 (46.67%) of T2 respondents reported having encountered a person at risk for suicide, from which *n* = 6 85.71% stated that they were able to intervene in all of the crises, and 14.29% (*n* = 1) described that there were instances in which they were able to intervene and others where they were unable because the brief window of time they have had with the person requiring support. Among the seven GKs who reported having intervened in a crisis, 13 interventions were carried out. The current article reports on the statistical findings of this mixed-methods study, and qualitative results will be published separately. However, the following narrative response, obtained at three-month follow-up, illustrates a GK’s experience applying course content during a real-world intervention:

I had better appreciation of noticing the initial warning signs. A calmness of approach when deciding how to respond to his messages, and what to suggest in terms of referral (I referred him to the Samaritans and told him to dial 999 if he is at risk of harming himself). I felt confident and in control; this, I feel strongly, is entirely down to the SIFA training I did.

The GKs’ feedback following their interventions indicates that from the sum of suicide intervention behaviours applied (*N* = 22), 50% (*n =* 11) involved identifying the warning signs for suicide, 27.27% (*n =* 6) represented the referral of a person at risk for suicide to further support, and 22.73% (*n* = 5) included approaching a person at risk for suicide. Interestingly, as seen in below in Table 2, GKs reported having identified a total of 25 warning signs for suicide that prompted them to intervene, equalling 14 of the original warning signs delivered during training.

**Table 2:** The total number of warning signs for suicide identified by gatekeepers in a three-month period following training

The Warning Signs for Suicide Included in The Suicide First Aid Guidelines for Supporting People from Migrant and refugee Backgrounds (*n =* 14)	Frequency of Identification (*N =* 25)
Hopelessness	4
Setting Affairs in Order	2
Feeling trapped, and that suicide is the only solution	3
Praying that God will take their life	1
Withdrawing from friends, family, or the community	1
Expressing that death is worth more than their life	1
Worthlessness	2
Threatening to hurt or kill themselves, or say that they wish to die, verbally, or in writing	4
Wanting to disappear	1
Drastic changes in mood, behaviour, or appearance	3
Isolation	1
Fear of deportation	2

Note: The discrepancy between frequency of identification (*N* = 25) and number of interventions (*N* = 13) was due to individuals who were supported often presenting more than one warning sign

## Discussion

The objectives of this study echo those that unite much of the existing GKT literature [44, 45]. These are to enhance knowledge, attitudes, self-efficacy, and skills to identify a person at risk of suicide, provide effective short-term support in a crisis, and facilitate the transition to further appropriate care. The key distinction, however, was that this study investigated knowledge about suicide that was deemed suitable to support people from AS&R backgrounds. Statistical analysis suggested that this GKT program was able to increase knowledge about suicide in multicultural contexts and that these improvements were able to be maintained three months following the completion of the program. The capacity for GKT to exert and maintain a positive influence over knowledge about suicide is a recurring outcome evident in existing literature [36, 46]. Notably, however, due to the scant evidence of suicide prevention efforts among people from AS&R backgrounds [26], relatively little is known about whether GKT can achieve this same impact among GKs’ who support people from these groups. Two particular studies resemble the current research and can serve as useful comparisons. The first, investigated effectiveness of GKT targeted at staff and volunteers who support people from AS&R backgrounds in Australia, concluding that they were able to enhance knowledge about suicide among GKs, which remained above baseline scores for six months [9]. The second incorporated the same guidelines used in the current study to pilot GKT for humanitarian workers who support children and young people in conflict-affected areas in Syria, which also observed a significant increase in knowledge about suicide [11]. However, the pilot project in Syria did not incorporate a long-term data collection point to measure the duration of those improvements. This study’s results, therefore, offer an original insight into how a GKT using the suicide guidelines as its knowledge base can increase knowledge about suicide with long-lasting effects.

The current research aimed to enhance GKs’ self-efficacy (SE) to intervene in a crisis, understand aspects of training that made this possible, and where the program could be modified to have a greater influence. Statistical analysis indicated that training was able to increase SE and that these improvements could be maintained three months following program completion. The ability of GKT to exert and maintain a positive influence over SE is a relatively consistent outcome demonstrated in previous program evaluation literature [38, 46, 47].

The statistical analysis conducted in this study indicates that training could not exert a quantifiable difference in GKs’ attitudes toward suicide and suicide prevention. Baseline scores demonstrate that GKs had positive attitudes towards suicide and suicide prevention before participating in training. Consequently, the two subsequent time points (i.e., immediately post-training and three-month follow-up) could not detect a meaningful increase in scores for this construct. Importantly, however, the inability to influence baseline scores for attitude is not uncommon in existing GKT evaluation literature, particularly when GKs’ attendance was voluntary. For instance, a GKT program delivered to community facilitators (i.e., teachers, nurses, social workers, pharmacists, clergy, counsellors, and carers for the elderly). Participants’ attitudes were generally positive when measured both pre-and post-training, leading to the inference that those who self-select to attend GKT already possess an intrinsic motivation to reduce suicide in their community. As a result, the researchers suggest that programs delivered to those who volunteer to participate should prioritise the development of knowledge and self-efficacy [48]. More recently, a systematic review of the long-term efficacy of GKT found an association between GKT investigations that targeted captive audiences (i.e., those who were obligated to attend) and significant changes in attitude towards suicide and suicide prevention; and non-significant changes when GKs chose to take part [31]. Consequently, the authors advise moving away from a universal approach to considering the context in which GKT is implemented, stating that focusing on participants’ attitudes towards suicide and suicide prevention may only be necessary for GKT programs delivered to non-captive audiences [31].

### Limitations

The absence of a control group may reduce the reliability of specific findings, as it is difficult to attribute, with any certainty, that within-group changes were the consequence of training, with no between-group comparison of training effects. Further, the current study suggests that training content was transferable to real-world suicide prevention behaviour. However, the ability of training to increase the rate of suicide prevention behaviour from a baseline measurement was not assessed. Therefore, it is not possible to assert that attendance at the course increased suicide prevention behaviour. The current research also included a small initial sample size compared to other GKT evaluations, which was further subject to a 50% attrition rate towards the end of the study. This may reduce the reliability of conclusions to be generalisable. Lastly, although the study appeared to positively influence outcome measures that could be maintained over a long period, the time between training and the follow-up surveys (three months) may account for the evident lack of deterioration.

### Future directions for research

The current research suggests that Suicide Intervention Training has a positive and lasting influence over knowledge about suicide and self-efficacy to intervene in a crisis. However, future research into this program or other GKT courses that incorporate the same guidelines could examine whether the improvements in these constructs can be maintained for 6 to 12 months post-training. Although previous research suggests that improvements in knowledge about suicide, attitudes towards suicide and prevention, and self-efficacy to intervene are associated with an increased likelihood of intervening in a crisis, little is known about how training content translates into real-world suicide prevention behaviour.

GKT research, in general, would benefit from studying the effects that training can have on actual suicide behaviours (i.e., thoughts, self-harm, attempts, and death by suicide), help-seeking behaviour, and quality of help provision. To understand these elements, future investigations could implement qualitative methods to explore the perspectives of those who have received an intervention from trained GKs.

While this study indicates that training was well-received in the United Kingdom. Subsequent research could explore whether using these guidelines to support people from asylum-seeking and refugee backgrounds in low- and middle-income countries, including their efficacy as standard or whether they need to be modified to increase their efficacy. Lastly, the realisation that training did not positively influence GKs’ attitudes toward suicide and prevention also highlights another consideration for future research into GKT. The notion that self-selected participation in training may accompany an intrinsic desire to prevent suicide and, therefore, correlate with adaptive attitudes towards suicide appears to be supported by this study. Accordingly, future research evaluating the efficacy of GKT among non-captive GKs may not need to examine the program’s ability to influence this outcome.

## Conclusions

The findings suggest that tailored suicide prevention training can have a significant influence on knowledge about suicide in multicultural contexts, self-efficacy to intervene in a crisis, and that course content translates effectively into real-world suicide prevention behaviour. Modifying program content appears to be a pivotal factor in promoting the support of people from asylum-seeking and refugee backgrounds. Lastly, following GKs’ positive appraisal of training, the organisation from which GKs were recruited to participate in this study requested that the program be integrated into the standard onboarding requirements of all new volunteers and employees.

## Data Availability

The data supporting this study’s findings are available on request from the corresponding author, SMH, by email: steven.macdonald-hart.ctr@usuhs.edu. Raw data are not publicly available as information may compromise the privacy of study participants.

## Footnotes

1 These guidelines must not be confused with the Suicide First Aid (SFA) training, a UK company that has trademarked this name. Although the label SFAG has been used for over a decade, the guidelines will be labelled suicide prevention guidelines to avoid confusion. Similarly, SIFA was used as an acronym before we became aware that SFA had been trademarked and refers to an intervention based on the freely-available SFAG and it is not linked to SFA UK. Intervention Training will be used to distinguish between program in this article and SFA the company.
